# Supplementary figures and images for: Sex differences in rates of permanent pacemaker implantation and in-hospital complications: A statewide cohort study of over 7 million persons from 2009–2018
Source: PLoS One. 2022 Aug 10;17(8):e0272305. doi: 10.1371/journal.pone.0272305 (PMC9365143; doi:10.1371/journal.pone.0272305)

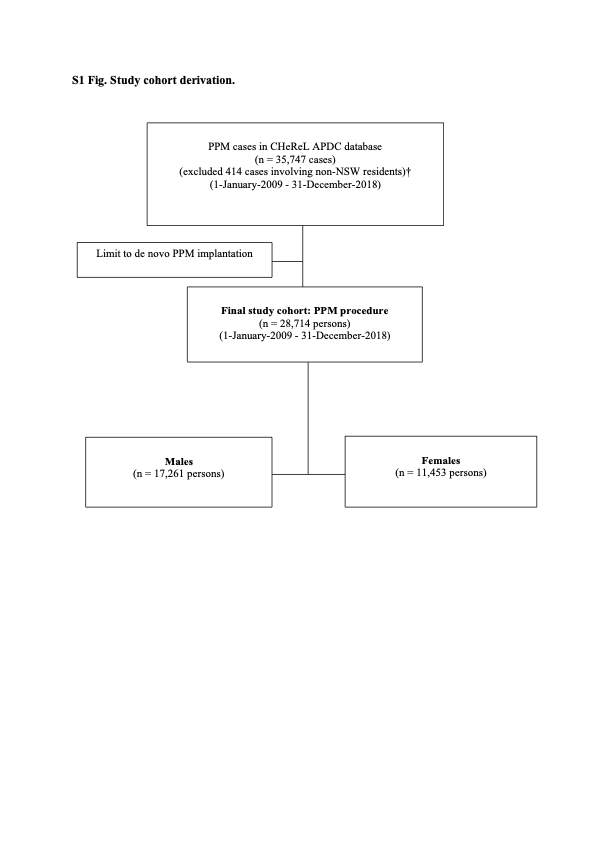

Supplement: S1 Fig — Flow chart shows the derivation of the study cohort. Abbreviations: APDC, Admitted Patient Data Collection; CHeReL, Centre for Health Record Linkage; NSW, New South Wales State of Australia; PPM, permanent pacemaker. (TIFF) [file pone.0272305.s001.tiff]

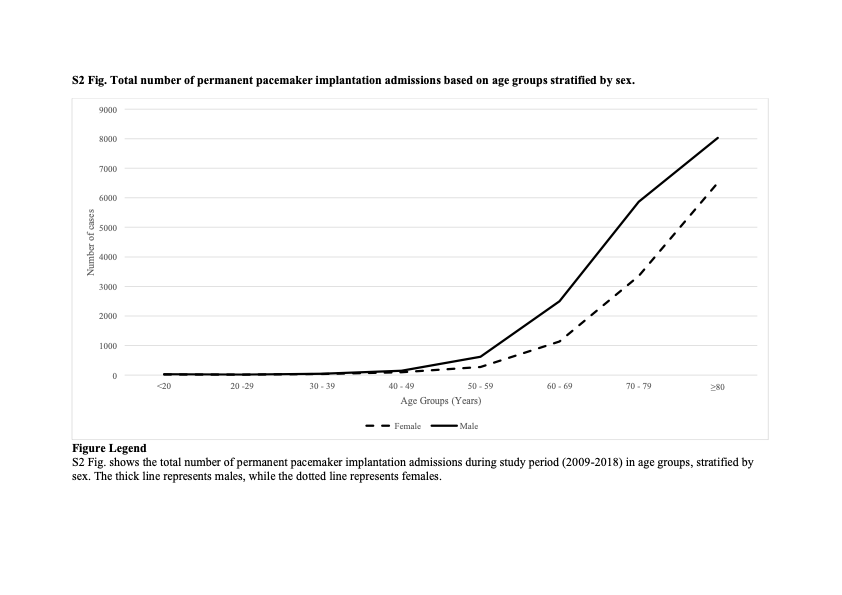

Supplement: S2 Fig — shows the total number of permanent pacemaker implantation admissions during study period (2009–2018) in age groups, stratified by sex. The thick line represents males, while the dotted line represents females. (TIFF) [file pone.0272305.s002.tiff]

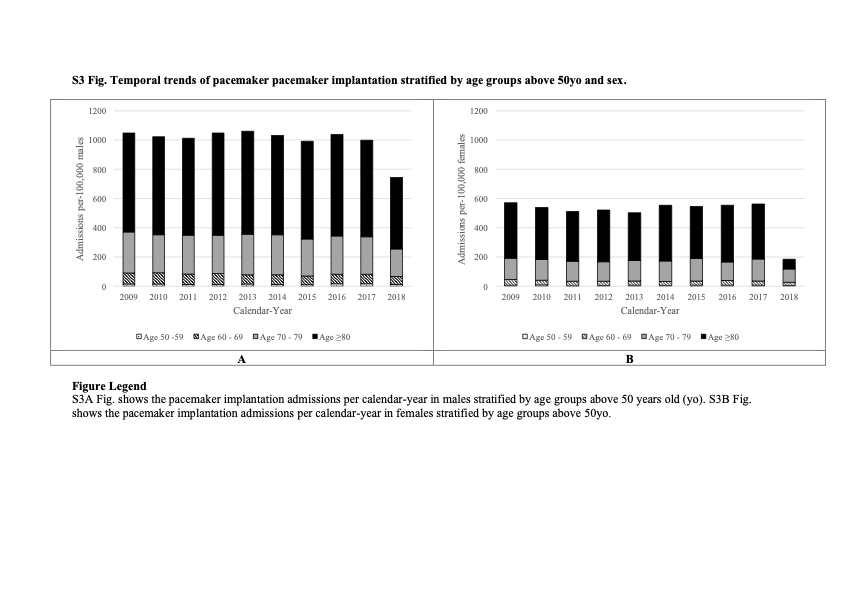

Supplement: S3 Fig — S3A Fig shows the pacemaker implantation admissions per calendar-year in males stratified by age groups above 50 years old (yo). S3B Fig shows the pacemaker implantation admissions per calendar-year in females stratified by age groups above 50yo. (TIFF) [file pone.0272305.s003.tiff]

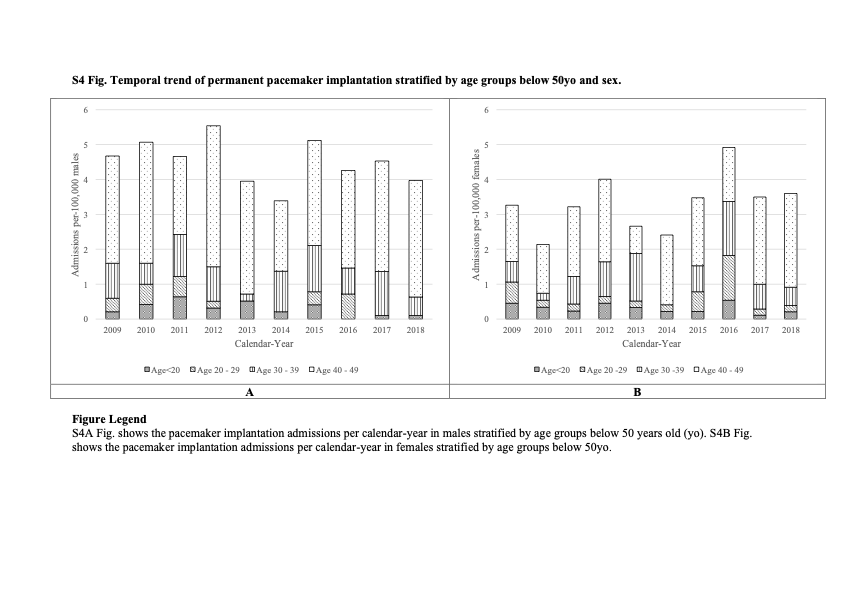

Supplement: S4 Fig — S4A Fig shows the pacemaker implantation admissions per calendar-year in males stratified by age groups below 50 years old (yo). S4B Fig shows the pacemaker implantation admissions per calendar-year in females stratified by age groups below 50yo. (TIFF) [file pone.0272305.s004.tiff]
