# Supplementary material for: Sex differences in rates of permanent pacemaker implantation and in-hospital complications: A statewide cohort study of over 7 million persons from 2009–2018
Source: PLoS One. 2022 Aug 10;17(8):e0272305. doi: 10.1371/journal.pone.0272305 (PMC9365143; doi:10.1371/journal.pone.0272305)
Supplement: S1 Table — (DOCX) [file pone.0272305.s005.docx]

**S1 Table. Study comorbidities and complications International Classification of Diseases Tenth Revision Australian Modification (ICD-10AM) codes and Australian Classification of Health Interventions (ACHI) procedural codes.**

| **No.** | **Comorbidity *** | **ICD-10AM codes** |
| --- | --- | --- |
| 1 | Indications for permanent pacemaker implantation | - Sick Sinus Syndrome (SSS): I49.5 - Complete Heart Block: I44.2 - Other AV block and bradycardia: I44.0, I44.1, I44.3, I44.4, I44.5, I44.6, I44.7, I45.0-I45.9, R00.1 |
| 2 | Atrial fibrillation/flutter | I48 |
| 3 | Acute myocardial infarction | I21, I22, I23 |
| 4 | Ischemic heart disease | I20, I21, I22, I23, I24, I25 |
| 5 | Prior percutaneous coronary intervention (PCI) / coronary artery bypass grafting (CABG) | Z95.1, Z95.5 |
| 6 | Congestive cardiac failure | I42, I43, I50, I11.0, I13.0, I13.2 |
| 7 | Valvular heart disease | I05, I06, I07, I08, I09.1, I09.8, I34, I35, I36, I37, I38, I39, Q22, Q23, Q24.87 |
| 8 | Peripheral vascular disease | E09.5, E10.51, E10.52, E11.51, E11.52, E13.51, E13.52, E14.51, E14.52, I70, I71, I72, I73, I74, I77, I78, I79 |
| 9 | Stroke | G45-45.9, G46-G46.8, I60, I61, I62, I63, I64 |
| 10 | Cardiovascular disease  (defined as morbidities item nos. 2, 4-10) | I48, I20-I25, Z95.1, Z95.5, I42, I43, I50, I11.0, I13.0, I13.2, I05, I06, I07, I08, I09.1, I09.8, I34, I35, I36, I37, I38, I39, Q22, Q23, Q24.87, I70, I71, I72, I73, I74, I77, I78, I79, E09.5, E10.51, E10.52, E11.51, E11.52, E13.51, E13.52, E14.51, E14.52, G45-45.9, G46-G46.8, I60-I62, I63-I64, Z95.2, Z95.3, Z95.4 |
| 11 | Hypertension | I10, I11, I12, I13, I15 |
| 12 | Hyperlipidemia | E78 |
| 13 | Diabetes | E09, E10, E11, E13, E14, Z92.22 |
| 14 | Current/ex-smoker | F17, Z72.0, Z86.43 |
| 15 | Cardiac risk factors  (defined as morbidities item nos. 12-15) | I10, I11, I12, I13, I15, E78, E09, E10, E11, E13, E14, Z92.22, Z72.0, F17, Z86.43 |
| 16 | Systemic connective tissue disease | M30, M31, M32, M33, M34, M35, M36 |
| 17 | Chronic pulmonary disease  (include asthma, chronic airways limitation, interstitial lung disease, cystic fibrosis with pulmonary manifestation) | E84.0, J40, J41, J42, J43, J44, J45, J46, J47, J60, J61, J62, J63, J64, J65, J66, J67, J68, J70, J82, J84, J99 |
| 18 | Malignancy | C00-C96, D00-D09 |
| 19 | Chronic kidney disease | N18, N19 |
| 20 | Dementia | F00, F01, F02, F03 |
| 21 | Neurodegenerative diseases  (defined as dementia, central nervous systemic atrophies, Parkinson’s disease, basal ganglia degeneration and/or nervous systemic degenerative diseases) | F00, F01, F02, F03, G10-G14, G20, G23, G30, G31 |
| 22 | Peptic ulcer disease | K25, K26, K27, K28 |
| 23 | Liver disease – mild | K70.0, K70.1, K70.2, K70.9, K71.0, K71.1, K71.2, K71.3, K71.4, K71.5, K71.6, K71.8, K71.9, K73, K75, K76, K77 |
| 24 | Liver disease – moderate-severe | I82.0, K70.3, K70.4, K71.7, K72, K74 |
| 25 | Chronic kidney disease – moderate-severe | N18.3, N18.4, N18.5 |
| 26 | Diabetes with organ damage | E09.21, E09.29, E09.31, E09.32, E09.40, E09.42, E09.51, E09.52, E09.71, E09.72, E09.8, E10.21, E10.22, E10.29, E10.31, E10.32, E10.33, E10.34, E10.35, E10.36, E10.39, E10.40, E10.41, E10.42, E10.43, E10.49, E10.51, E10.52, E10.53, E10.61, E10.62, E10.63, E10.69, E10.71, E10.73, E10.8, E11.21, E11.22, E11.29, E11.31, E11.32, E11.33, E11.34, E11.35, E11.36, E11.39, E11.40, E11.41, E11.42, E11.43, E11.49, E11.51, E11.52, E11.53, E11.61, E11.62, E11.63, E11.69, E11.71, E11.72, E11.73, E11.8, E13.21, E13.22, E13.29, E13.31, E13.32, E13.33, E13.34, E13.35, E13.36, E13.39, E13.40, E13.41, E13.42, E13.43, E13.49, E13.51, E13.52, E13.53, E13.61, E13.62, E13.63, E13.69, E13.71, E13.72, E13.73, E13.8, E14.21, E14.22, E14.29, E14.31, E14.32, E14.33, E14.34, E14.35, E14.36, E14.39, E14.40, E14.41, E14.42, E14.43, E14.49, E14.51, E14.52, E14.53, E14.61, E14.62, E14.63, E14.69, E14.71, E14.72, E14.73, E14.8 |
| 27 | Lymphoma | C81, C82, C83, C84, C85, C86, C88 |
| 28 | Leukemia | C90, C91, C92, C93, C94, C95, C96 |
| 29 | Metastatic solid tumor | C76, C77, C78, C79, C80 |
| 30 | Hemiplegia | G81, G82 |
| 31 | Acquired Immune Deficiency Syndrome  (AIDS) | B20, B21, B22, B23, B24 |
| 32 | Any tumor/malignancy excluding lymphoma and/or leukemia | C00-C80, D00-D09 |
| 33 | Cx_VTEpostProcDuringAdmission  (Defined as BD_VTE AND concomitantly include T82.8) | I26, I26.0, I26.9, I80, I80.0, I80.1, I80.2, I80.3, I80.8, I80.9, I81, I82, I82.0, I82.1, I82.2, I82.3, I82.8, I82.9, T82.8 |
| 34 | Cx_DVTpostProcDuringAdmission  (Defined as BD_DVT AND concomitantly include T82.8) | I80, I80.0, I80.1, I80.2, I80.3, I80.8, I80.9, I81, I82, I82.0, I82.1, I82.2, I82.3, I82.8, I82.9, T82.8 |
| 35 | Cx_PEpostProcDuringAdmission  (Defined as BD_PE AND concomitantly include T82.8) | I26, I26.0, I26.9, T82.8 |
| 36 | Cx_OtherCxpostCardVascDevices  (include embolism/fibrosis/haemorrhage/pain/stenosis/thrombosis) | T82.8 |
| 37 | Cx_InfectionpostProc | T81.41, T81.42 |
| 38 | Cx_InfectionpostCardVascDevices | T82.7 |
| 39 | Cx_PneumothoraxDuringAdmission | J93, J93.8, J93.9, J931, J938, J939 |
| 40 | Cx_Haemathorax | J942 |
| 41 | Cx_InjuriesofHeart | S26, S26.8, S26.81, S26.82, S26.83, S26.88, S26.9 |
| 42 | Cx_Cardiac tamponade | I31.9 |
| 43 | Cx_Haemopericardium | I31.2 |
| 44 | Cx_Pericardialeffusion | I31.3 |
| 45 | Cx_MechanicalcomplicationCIED | T82.1 |
| BD, background diagnosis; CIED, cardiac implantable electronic device; Cx, complication; DVT, deep vein thrombosis; PE, pulmonary embolism; VTE, venous thromboembolism.   - To calculate the Charlson Comorbidity Index (CCI) score, without age adjustment, for individual patient during a particular admission of interest, use the following morbidity item numbers with their corresponding ICD-10AM codes to derive the patient’s CCI score:   1. 1 score for each morbidity item – 3, 6, 8, 9, 13, 16, 17, 20, 22, 23   2. 2 score for each morbidity item – 25, 26, 27, 28, 30, 32   3. 3 score for morbidity item – 24   4. 6 score for morbidity item – 29, 31 | | |

| **No.** | **ACHI procedures** | **ACHI procedural codes** |
| --- | --- | --- |
| 1 | Procedure_permanent pacemaker implantation | 38353-00 |
| 2 | Cx_PocketComplication_Revision procedures on other sites of the heart that includes debridement | 90219-00 |
| 3 | Cx_LeadManipulation_Adjustment of epicardial electrode for cardiac pacemaker (includes repair/ repositioning/ revision and stabilisation) of epicardial electrode of permanent pacemaker (includes that via sternotomy/ subxiphoid approach/ thoracotomy); excludes that with defibrillator functionality | 38456-21 |
| 4 | Cx_LeadManipulation_Adjustment of transvenous electrode for cardiac pacemaker – includes repair/ repositioning/ revision and stabilisation | 90203-00 |
| 5 | Cx_GeneratorManipulation_Adjustment, replacement or removal of cardiac pacemaker generator repair, repositioning or revision of the generator | 90203-05 |
| Cx, complication | | |
