# Supplementary material for: Sex differences in rates of permanent pacemaker implantation and in-hospital complications: A statewide cohort study of over 7 million persons from 2009–2018
Source: PLoS One. 2022 Aug 10;17(8):e0272305. doi: 10.1371/journal.pone.0272305 (PMC9365143; doi:10.1371/journal.pone.0272305)
Supplement: S2 Table — (DOCX) [file pone.0272305.s006.docx]

**S2 Table. Permanent pacemaker implantation in-hospital complications stratified by sex (based on all admissions involving permanent pacemaker implantations during study period).**

| **Complications, no. (%)** | **Total cases**  **(n=35,747)** | **Stratified by sex** | | **P value** |
| --- | --- | --- | --- | --- |
|  |  | **Males**  **(n=21,471; 60.1%)** | **Females**  **(n= 14,276; 39.9%)** |  |
| Total non-fatal complications | 2,339 (6.54) | 1,286 (6.00) | 1,053 (7.38) | <0.001 |
| Venous thromboembolism | 55 (0.15) | 28 (0.13) | 27 (0.19) | 0.17 |
| Pulmonary embolism | 9 (0.02) | 3 (0.01) | 6 (0.04) | 0.17 |
| Deep venous thrombosis | 48 (0.13) | 26 (0.12) | 22 (0.15) | 0.46 |
| Infection post-implantation | 256 (0.72) | 159 (0.74) | 97 (0.68) | 0.52 |
| Pocket complications | 8 (0.02) | 6 (0.03) | 2 (0.01) | 0.49 |
| Cardiac injuries | 40 (0.11) | 12 (0.06) | 28 (0.20) | <0.01 |
| Pericardial effusion | 198 (0.55) | 89 (0.41) | 109 (0.76) | <0.01 |
| Cardiac tamponade | 63 (0.18) | 24 (0.11) | 39 (0.27) | <0.01 |
| Pneumothorax | 361 (1.00) | 167 (0.78) | 194 (1.36) | <0.01 |
| Haemothorax | 0 (0) | 0 (0) | 0 (0) | NA |
| Hemopericardium | 5 (0.01) | 3 (0.01) | 2 (0.01) | 1.00 |
| Mechanical complications* | 514 (1.44) | 303 (1.41) | 211 (1.48) | 0.62 |
| Lead manipulation | 399 (1.12) | 238 (1.11) | 161 (1.13) | 0.88 |
| Generator manipulation | 24 (0.07) | 15 (0.07) | 9 (0.06) | 1.00 |
| Others† | 723 (2.02) | 404 (1.88) | 319 (2.23) | 0.02 |
| Values represent number of patients with values in brackets representing percentages, or otherwise stated.  NA, not applicable.   - Defined as breakdown, displacement, malposition, leakage, obstruction, perforation or protrusion. - Include embolisms, fibrosis, haemorrhage, pain, stenosis or thrombosis. | | | | |
