# Supplementary material for: Sex differences in rates of permanent pacemaker implantation and in-hospital complications: A statewide cohort study of over 7 million persons from 2009–2018
Source: PLoS One. 2022 Aug 10;17(8):e0272305. doi: 10.1371/journal.pone.0272305 (PMC9365143; doi:10.1371/journal.pone.0272305)
Supplement: S3 Table — (DOCX) [file pone.0272305.s007.docx]

**S3 Table. Univariable predictors for total in-hospital non-fatal complications.**

| **Parameters** | **Odds ratio (95% CI)** | **P value** |
| --- | --- | --- |
| Males | 0.80 (0.73 – 0.87) | <0.001 |
| Year of admission |  | <0.001 |
| 2009 | 1.00 (reference) |  |
| 2010 | 0.92 (0.75 – 1.11) | 0.39 |
| 2011 | 0.86 (0.70 – 1.05) | 0.14 |
| 2012 | 0.94 (0.77 – 1.14) | 0.51 |
| 2013 | 0.86 (0.71 – 1.05) | 0.15 |
| 2014 | 1.07 (0.89 – 1.29) | 0.48 |
| 2015 | 1.07 (0.88 – 1.23) | 0.51 |
| 2016 | 0.99 (0.82 – 1.20) | 0.94 |
| 2017 | 0.69 (0.56 – 0.84) | <0.001 |
| 2018 | 0.47 (0.37 – 0.60) | <0.001 |
| Age – per 1-year increase | 0.99 (0.98 – 0.99) | <0.001 |
| Referral source |  | <0.001 |
| Emergency department | 1.00 (reference) |  |
| Elective | 0.53 (0.48 – 0.59) | <0.001 |
| External hospital-referred | 0.71 (0.63 – 0.80) | <0.001 |
| Others | 0.53 (0.32 – 0.90) | <0.01 |
| Unknown | 1.03 (0.55 – 1.93) | 0.93 |
| Type of facility |  |  |
| Public | 1.00 (reference) |  |
| Private | 0.58 (0.53 – 0.63) | <0.001 |
| Indications for PPM |  |  |
| Complete heart block | 1.63 (1.47 – 1.80) | <0.001 |
| Other AV block and bradycardia | 1.04 (0.95 – 1.14) | 0.38 |
| Sick sinus syndrome | 0.83 (0.75 – 0.93) | <0.001 |
| Others | 0.91 (0.81 – 1.02) | 0.12 |
| Other primary diagnosis |  |  |
| Acute coronary syndrome | 2.24 (1.78 – 2.83) | <0.001 |
| Concomitant cardiac procedures during admission | |  |
| CABG | 3.63 (2.96 – 4.46) | <0.001 |
| All cardiac valve surgery | 4.51 (3.87 – 5.26) | <0.001 |
| TAVI | 4.07 (2.63 – 6.28) | <0.001 |
| Ischaemic heart disease | 1.76 (1.57 – 2.00) | <0.001 |
| Prior PCI / CABG | 1.14 (0.96 – 1.35) | 0.14 |
| Congestive cardiac failure | 2.12 (1.86 – 2.42) | <0.001 |
| Stroke | 2.44 (1.80 – 3.31) | <0.001 |
| Peripheral vascular disease | 2.22 (1.78 – 2.74) | <0.001 |
| Valvular heart disease | 2.95 (2.52 – 3.46) | <0.001 |
| Prosthetic heart valve | 1.28 (0.95 – 1.73) | 0.11 |
| Atrial fibrillation/flutter | 1.70 (1.55 – 1.87) | <0.001 |
| Hypertension | 1.76 (1.60 – 1.94) | <0.001 |
| Hyperlipidemia | 1.14 (0.83 – 1.56) | 0.43 |
| Diabetes | 1.01 (0.96 – 1.20) | 0.19 |
| Current/ex-smoker | 0.93 (0.84 – 1.02) | 0.13 |
| Malignancy | 3.93 (2.86 – 5.41) | <0.001 |
| Chronic pulmonary disease | 2.73 (2.19 – 3.41) | <0.001 |
| Chronic kidney disease | 2.05 (1.78 – 2.35) | <0.001 |
| CCI score – per 1-score * | 1.20 (1.16 – 1.22) | <0.001 |
| AV, atrioventricular; CABG, coronary artery bypass graft; TAVI, transaortic valve intervention; CCI, Charlson comorbidity index; CI, confidence interval; OR, odds ratio; PCI, percutaneous coronary interventions; PPM, permanent   - Conditions included in the Charlson Comorbidity Index include myocardial infarction, congestive cardiac failure, peripheral vascular disease, stroke, dementia, chronic pulmonary disease, connective tissue disease, peptic ulcer disease, liver disease (mild vs. moderate to severe), diabetes (with or without organ damage), hemiplegia, moderate to severe renal disease, any tumour (within last 5 years), lymphoma, leukaemia, metastatic solid tumour and acquired immunodeficiency syndrome (AIDS). | | |
