# Supplementary material for: Sex differences in rates of permanent pacemaker implantation and in-hospital complications: A statewide cohort study of over 7 million persons from 2009–2018
Source: PLoS One. 2022 Aug 10;17(8):e0272305. doi: 10.1371/journal.pone.0272305 (PMC9365143; doi:10.1371/journal.pone.0272305)
Supplement: S4 Table — (DOCX) [file pone.0272305.s008.docx]

**S4 Table. Predictors for in-hospital death.**

| **Parameters** | **Univariable OR** | **P value** | **Multivariable OR*** | **P value** |
| --- | --- | --- | --- | --- |
| Males | 0.89 (0.68 – 1.18) | 0.42 |  |  |
| Year of admission |  | 0.84 |  |  |
| 2009 | 1.00 (reference) |  |  |  |
| 2010 | 1.14 (0.57 – 2.30) | 0.71 |  |  |
| 2011 | 1.49 (0.77 – 2.89) | 0.23 |  |  |
| 2012 | 1.29 (0.66 – 2.53) | 0.46 |  |  |
| 2013 | 1.47 (0.77 – 2.82) | 0.25 |  |  |
| 2014 | 1.23 (0.63 – 2.40) | 0.55 |  |  |
| 2015 | 1.54 (0.81 – 2.93) | 0.19 |  |  |
| 2016 | 1.12 (0.56 – 2.18) | 0.77 |  |  |
| 2017 | 1.65 (0.89 – 3.09) | 0.12 |  |  |
| 2018 | 1.46 (0.74 – 2.88) | 0.28 |  |  |
| Age – per 1-year increase | 1.00 (0.98 – 1.01) | 0.68 |  |  |
| Referral source |  | <0.001 |  | 0.02 |
| Emergency department | 1.00 (reference) | <0.001 | 1.00 (reference) |  |
| Elective | 0.22 (0.15 – 0.31) | <0.001 | 0.48 (0.32 – 0.73) | <0.001 |
| External hospital-referred | 0.56 (0.41 – 0.77) | <0.001 | 0.75 (0.53 – 1.07) | 0.11 |
| Others | 0.50 (0.12 – 2.03) | 0.33 | 0.71 (0.17 – 2.99) | 0.64 |
| Unknown | 0.67 (0.09 – 4.82) | 0.69 | 0.68 (0.08 – 5.73) | 0.72 |
| Type of facility |  |  |  |  |
| Public | 1.00 (reference) |  | 1.00 (reference) |  |
| Private | 0.37 (0.27 – 0.51) | <0.001 | 0.78 (0.55– 1.11) | 0.17 |
| Indications for PPM |  |  |  |  |
| Complete heart block | 3.00 (2.27 – 3.96) | <0.001 | 1.73 (1.27 – 2.36) | <0.001 |
| Other AV block and bradycardia | 1.10 (0.84 – 1.44) | 0.51 |  |  |
| Sick sinus syndrome | 0.45 (0.30 – 0.66) | <0.001 | 0.58 (0.38 – 0.89) | 0.01 |
| Others | 0.80 (0.55 – 1.16) | 0.24 |  |  |
| Other primary diagnosis |  |  |  |  |
| Acute coronary syndrome | 0.22 (0.14 – 0.36) | <0.001 | 1.39 (0.79 – 2.46) | 0.25 |
| Concomitant procedures during admission | |  |  |  |
| CABG | 2.50 (1.32 – 4.74) | 0.005 | 0.42 (0.20 – 0.88) | 0.02 |
| All cardiac valve surgery | 5.25 (3.58 – 7.70) | <0.001 | 2.11 (1.28 – 3.48) | 0.004 |
| TAVI | 1.22 (0.17 – 8.77) | 0.844 |  |  |
| Ischemic heart disease | 4.20 (3.17 - 5.57) | <0.001 | 2.14 (1.54 – 2.97) | <0.001 |
| Prior PCI / CABG | 1.03 (0.63 – 1.70) | 0.91 |  |  |
| Congestive cardiac failure | 9.36 (7.10 – 12.4) | <0.001 | 3.60 (2.65 – 4.89) | <0.001 |
| Stroke | 11.3 (7.13 – 17.8) | <0.001 | 5.95 (3.59 – 9.86) | <0.001 |
| Peripheral vascular disease | 5.30 (3.47 – 8.11) | <0.001 | 2.19 (1.37 – 3.51) | 0.001 |
| Valvular heart disease | 5.05 (3.51 – 7.27) | <0.001 | 1.45 (0.94 – 2.23) | 0.10 |
| Prosthetic heart valve | 1.30 (0.41 – 4.07) | 0.65 |  |  |
| Atrial fibrillation/flutter | 3.04 (2.32 – 4.00) | <0.001 | 2.09 (1.54 – 2.83) | <0.001 |
| Hypertension | 2.83 (2.15 – 3.74) | <0.001 | 1.02 (0.75 – 1.39) | 0.91 |
| Hyperlipidemia | 1.07 (0.39 – 2.88) | 0.90 |  |  |
| Diabetes | 1.74 (1.29 – 2.35) | <0.001 | 0.83 (0.60 – 1.17) | 0.29 |
| Current/ex-smoker | 1.18 (0.88 – 1.57) | 0.28 |  |  |
| Malignancy | 8.45 (4.64 – 15.38) | <0.001 | 4.44 (2.29 – 8.61) | <0.001 |
| Chronic pulmonary disease | 7.28 (4.78 – 11.07) | <0.001 | 2.30 (1.45 – 3.63) | <0.001 |
| Chronic kidney disease | 8.10 (6.09 – 10.77) | <0.001 | 3.48 (2.51 – 4.83) | <0.001 |
| AV, atrioventricular; CABG, coronary artery bypass graft; CI, confidence interval; OR, odds ratio; PCI, percutaneous coronary interventions; PPM, permanent, TAVI, transcutaneous aortic valve implantation.   - Multivariable binary logistic regression method was used to identify independent predictors for in-hospital death; only univariables with P<0.05 were included in the multivariable analysis. | | | | |
