# Supplementary material for: Sex differences in rates of permanent pacemaker implantation and in-hospital complications: A statewide cohort study of over 7 million persons from 2009–2018
Source: PLoS One. 2022 Aug 10;17(8):e0272305. doi: 10.1371/journal.pone.0272305 (PMC9365143; doi:10.1371/journal.pone.0272305)
Supplement: S5 Table — (DOCX) [file pone.0272305.s009.docx]

**S5 Table. In-hospital cause-specific death during admission for permanent pacemaker implantation.**

| **Cause of death** | **In hospital, no (%)*** | | | |
| --- | --- | --- | --- | --- |
|  | **Total cohort†** | **Males ‡** | **Females ‡** | **P value §** |
| **Cardiovascular** | **107 (51.2)** | **58 (48.3)** | **49 (55.1)** | 0.46 |
| Pulmonary embolism | 6 (2.9) | 4 (3.3) | 2 (2.2) |  |
| Cardiac arrest | 3 (1.4) | 1 (0.8) | 2 (2.2) |  |
| Acute myocardial infarction | 12 (5.7) | 6 (5.0) | 6 (6.7) |  |
| Heart failure | 46 (22.0) | 30 (25.0) | 16 (18.0) |  |
| Cardiac-related ‖ | 31 (14.8) | 14 (11.7) | 17 (19.1) |  |
| Embolic stroke | 6 (2.9) | 3 (2.5) | 3 (3.4) |  |
| Haemorrhagic stroke | 3 (1.4) | 0 (0) | 3 (3.4) |  |
| Stroke-related ‖ | 0 (0) | 0 (0) | 0 (0) |  |
| **Noncardiovascular** | **102 (48.8)** | **62 (51.7)** | **40 (44.9)** |  |
| Sepsis | 38 (18.2) | 29 (24.2) | 9 (10.1) |  |
| Malignancy | 2 (1.0) | 1 (0.8) | 1 (1.1) |  |
| Other | 23 (11.0) | 10 (8.3) | 13 (14.6) |  |
| Undefined | 39 (18.7) | 22 (18.3) | 17 (19.1) |  |
| - No. (%) represents total number of deaths from each specific cause and value in brackets represents the percentage out of total deaths in each studied cohort (i.e. total cohort, male vs female groups). - There was a total of 209 in-hospital deaths for the total cohort. - There was a total of 120 in-hospital deaths in male vs 89 in female. - P value represents comparison based on chi square analysis between males and females. - Cardiac-related cause of death is coded when more than one cardiac cause of death is recorded on the death certificate; similarly, stroke-related cause of death is coded when more than one stroke cause of death (i.e. both embolic and haemorrhagic stroke) is recorded on the death certificate. | | | | |
