# Supplementary material for: Sex differences in rates of permanent pacemaker implantation and in-hospital complications: A statewide cohort study of over 7 million persons from 2009–2018
Source: PLoS One. 2022 Aug 10;17(8):e0272305. doi: 10.1371/journal.pone.0272305 (PMC9365143; doi:10.1371/journal.pone.0272305)
Supplement: S6 Table — (DOCX) [file pone.0272305.s010.docx]

**S6 Table. Independent predictors for total in-hospital non-fatal complications (multivariable model not including CCI)***

| **Parameters** | **Odds ratio (95% CI)** | **P value** |
| --- | --- | --- |
| Males | 0.78 (0.71 – 0.86) | <0.001 |
| Year of admission |  | <0.001 |
| 2009 | 1.00 (reference) |  |
| 2010 | 0.91 (0.75 – 1.12) | 0.38 |
| 2011 | 0.88 (0.72 – 1.08) | 0.23 |
| 2012 | 0.93 (0.76 – 1.14) | 0.49 |
| 2013 | 0.86 (0.70 – 1.05) | 0.13 |
| 2014 | 1.03 (0.85 – 1.25) | 0.79 |
| 2015 | 1.04 (0.86 – 1.27) | 0.66 |
| 2016 | 1.02 (0.84 – 1.23) | 0.88 |
| 2017 | 0.66 (0.54 – 0.82) | <0.001 |
| 2018 | 0.41 (0.32 – 0.53) | <0.001 |
| Age – per 1-year increase | 0.99 (0.98 – 1.00) | <0.001 |
| Referral source |  | <0.001 |
| Emergency department | 1.00 (reference) |  |
| Elective | 0.64 (0.57– 0.73) | <0.001 |
| External hospital-referred | 0.80 (0.71 – 0.91) | <0.001 |
| Others | 0.64 (0.38 – 1.07) | 0.90 |
| Unknown | 0.86 (0.45 – 1.64) | 0.64 |
| Type of facility |  |  |
| Public | 1.00 (reference) |  |
| Private | 0.77 (0.69 – 0.85) | <0.001 |
| Complete heart block | 1.20 (1.07 – 1.34) | 0.002 |
| Sick sinus syndrome | 0.92 (0.82 – 1.03) | 0.14 |
| Acute coronary syndrome | 1.28 (0.98 – 1.66) | 0.07 |
| CABG | 1.36 (1.05 – 1.76) | 0.02 |
| All cardiac valve surgery | 2.75 (2.26 – 3.34) | <0.001 |
| TAVI | 5.39 (3.38 – 8.58) | <0.001 |
| Ischaemic heart disease | 1.14 (0.99 – 1.30) | 0.06 |
| Congestive cardiac failure | 1.28 (1.11 – 1.47) | <0.001 |
| Stroke | 1.45 (1.05 – 2.01) | <0.001 |
| Peripheral vascular disease | 1.47 (1.18 – 1.84) | <0.001 |
| Valvular heart disease | 1.48 (1.23 – 1.77) | <0.001 |
| Atrial fibrillation/flutter | 1.30 (1.17 – 1.45) | <0.001 |
| Hypertension | 1.16 (1.04 – 1.30) | 0.01 |
| Malignancy | 3.11 (2.23 – 4.35) | <0.001 |
| Chronic pulmonary disease | 1.61 (1.27 – 2.03) | <0.001 |
| Chronic kidney disease | 1.25 (1.07 – 1.46) | 0.004 |
| CABG, coronary artery bypass graft; CCI, Charlson comorbidity index; CI, confidence interval; TAVI, transcutaneous aortic valve implantation.   - Multivariable binary logistic regression method was used to identify independent predictors for all in-hospital complications; only univariables with P<0.05 were included in the multivariable analysis. | | |
