# Supplementary material for: Sex differences in rates of permanent pacemaker implantation and in-hospital complications: A statewide cohort study of over 7 million persons from 2009–2018
Source: PLoS One. 2022 Aug 10;17(8):e0272305. doi: 10.1371/journal.pone.0272305 (PMC9365143; doi:10.1371/journal.pone.0272305)
Supplement: S8 Table — (DOCX) [file pone.0272305.s012.docx]

**S8 Table. Independent predictors for total in-hospital non-fatal complications (multivariable model including CCI) * in women.**

| **Parameters** | **Odds ratio (95% CI)** | **P value** |
| --- | --- | --- |
| Age – per 1-year increase | 0.98 (0.978 – 0.99) | <0.001 |
| Year of admission |  | <0.001 |
| 2009 | 1.00 (reference) |  |
| 2010 | 0.86 (0.64 – 1.15) | 0.31 |
| 2011 | 0.94 (0.70 – 1.27) | 0.70 |
| 2012 | 0.92 (0.69 – 1.24) | 0.59 |
| 2013 | 0.88 (0.65 – 1.18) | 0.38 |
| 2014 | 0.93 (0.70 – 1.23) | 0.59 |
| 2015 | 0.83 (0.62 – 1.11) | 0.21 |
| 2016 | 1.03 (0.78 – 1.37) | 0.82 |
| 2017 | 0.64 (0.47 – 0.86) | <0.01 |
| 2018 | 0.35 (0.24 – 0.52) | <0.001 |
| Referral source |  | <0.05 |
| Emergency department | 1.00 (reference) |  |
| Elective | 0.73 (0.60 – 0.88) | <0.001 |
| External hospital-referred | 0.89 (0.74 – 1.07) | 0.21 |
| Others | 0.73 (0.33 – 1.61) | 0.44 |
| Unknown | 1.04 (0.40 – 2.73) | 0.94 |
| Type of facility |  |  |
| Public | 1.00 (reference) |  |
| Private | 0.75 (0.64 – 0.88) | <0.001 |
| Complete heart block | 1.33 (1.10 – 1.60) | <0.01 |
| Sick sinus syndrome | 0.96 (0.81 – 1.15) | 0.68 |
| Others | 0.91 (0.74 – 1.13) | 0.39 |
| Acute coronary syndrome | 1.37 (0.91 – 2.05) | 0.13 |
| CABG | 0.95 (0.58 – 1.54) | 0.82 |
| All cardiac valve surgery | 2.23 (1.65 – 3.03) | <0.001 |
| TAVI | 4.81 (2.43 – 9.51) | <0.001 |
| Valvular heart disease | 1.40 (1.05 – 1.85) | <0.05 |
| Prosthetic heart valve | 1.58 (1.01 – 2.47) | 0.046 |
| Atrial fibrillation/flutter | 1.36 (1.16 – 1.59) | <0.001 |
| Hypertension | 1.22 (1.04 – 1.43) | <0.05 |
| CCI score – per 1-score † | 1.11 (1.07 – 1.16) | <0.001 |
| CABG, coronary artery bypass graft; TAVI, transcutaneous aortic valve implantation; CCI, Charlson comorbidity index; CI, confidence interval.   - Multivariable binary logistic regression method was used to identify independent predictors for all in-hospital complications; only univariables with P<0.05 were included in the multivariable analysis (refer to Supplementary Table 7 for univariable analysis). - Conditions included in the Charlson Comorbidity Index include myocardial infarction, congestive cardiac failure, peripheral vascular disease, stroke, dementia, chronic pulmonary disease, connective tissue disease, peptic ulcer disease, liver disease (mild vs. moderate to severe), diabetes (with or without organ damage), hemiplegia, moderate to severe renal disease, any tumor (within last 5 years), lymphoma, leukemia, metastatic solid tumor and acquired immunodeficiency syndrome (AIDS). | | |
