# Supplementary material for: Sex differences in rates of permanent pacemaker implantation and in-hospital complications: A statewide cohort study of over 7 million persons from 2009–2018
Source: PLoS One. 2022 Aug 10;17(8):e0272305. doi: 10.1371/journal.pone.0272305 (PMC9365143; doi:10.1371/journal.pone.0272305)
Supplement: S9 Table — (DOCX) [file pone.0272305.s013.docx]

**S9 Table. Independent predictors for total in-hospital non-fatal complications (multivariable model not including CCI) * in women.**

| **Parameters** | **Odds ratio (95% CI)** | **P value** |
| --- | --- | --- |
| Age – per 1-year increase | 0.98 (0.98 – 0.99) | <0.001 |
| Year of admission |  | <0.001 |
| 2009 | 1.00 (reference) |  |
| 2010 | 0.84 (0.62 – 1.13) | 0.24 |
| 2011 | 0.91 (0.68 – 1.23) | 0.53 |
| 2012 | 0.92 (0.69 – 1.23) | 0.57 |
| 2013 | 0.87 (0.65 – 1.18) | 0.36 |
| 2014 | 0.94 (0.71 – 1.25) | 0.67 |
| 2015 | 0.85 (0.64 – 1.14) | 0.28 |
| 2016 | 1.05 (0.79 – 1.39) | 0.74 |
| 2017 | 0.65 (0.48 – 0.88) | 0.01 |
| 2018 | 0.36 (0.24 – 0.53) | <0.001 |
| Referral source |  | 0.02 |
| Emergency department | 1.00 (reference) |  |
| Elective | 0.72 (0.60 – 0.88) | <0.001 |
| External hospital-referred | 0.89 (0.74 – 1.07) | 0.23 |
| Others | 0.73 (0.33 – 1.61) | 0.43 |
| Unknown | 1.06 (0.41 – 2.77) | 0.90 |
| Type of facility |  |  |
| Public | 1.00 (reference) |  |
| Private | 0.73 (0.62 – 0.85) | <0.001 |
| Complete heart block | 1.31 (1.08 – 1.58) | 0.01 |
| Sick sinus syndrome | 0.96 (0.80 – 1.14) | 0.62 |
| Others | 0.91 (0.73 – 1.12) | 0.36 |
| Acute coronary syndrome | 1.33 (0.87 – 2.02) | 0.18 |
| CABG | 0.99 (0.60 – 1.64) | 0.96 |
| All cardiac valve surgery | 2.15 (1.58 – 2.93) | <0.001 |
| TAVI | 4.34 (2.16 – 8.70) | <0.001 |
| Ischaemic heart disease | 1.05 (0.84 – 1.32) | 0.64 |
| Congestive cardiac failure | 1.23 (0.99 – 1.52) | 0.06 |
| Stroke | 1.34 (0.82 – 2.19) | 0.25 |
| Peripheral vascular disease | 2.33 (1.63 – 3.31) | <0.001 |
| Valvular heart disease | 1.37 (1.03 – 1.82) | 0.03 |
| Prosthetic heart valve | 1.54 (0.98 – 2.41) | 0.06 |
| Atrial fibrillation/flutter | 1.33 (1.13 – 1.55) | <0.001 |
| Hypertension | 1.24 (1.05 – 1.46) | 0.01 |
| Diabetes | 0.91 (0.75 – 1.10) | 0.33 |
| Malignancy | 3.30 (1.90 – 5.75) | <0.001 |
| Chronic pulmonary disease | 1.20 (0.78 – 1.85) | 0.41 |
| Chronic kidney disease | 1.23 (0.96 – 1.57) | 0.10 |
| CABG, coronary artery bypass graft; CI, confidence interval; TAVI, transcutaneous aortic valve implantation.   - Multivariable binary logistic regression method was used to identify independent predictors for all in-hospital complications; only univariables with P<0.05 were included in the multivariable analysis. | | |
