# Supplementary material for: Sex differences in rates of permanent pacemaker implantation and in-hospital complications: A statewide cohort study of over 7 million persons from 2009–2018
Source: PLoS One. 2022 Aug 10;17(8):e0272305. doi: 10.1371/journal.pone.0272305 (PMC9365143; doi:10.1371/journal.pone.0272305)
Supplement: S12 Table — (DOCX) [file pone.0272305.s016.docx]

**S12 Table. Independent predictors for total in-hospital non-fatal complications (multivariable model not including CCI) * in men.**

| **Parameters** | **Odds ratio (95% CI)** | **P value** |
| --- | --- | --- |
| Age – per 1-year increase | 0.992 (0.987 – 0.998) | <0.01 |
| Year of admission |  | <0.001 |
| 2009 | 1.00 (reference) |  |
| 2010 | 0.99 (0.75 – 1.30) | 0.91 |
| 2011 | 0.86 (0.64 – 1.14) | 0.29 |
| 2012 | 0.94 (0.71 – 1.24) | 0.65 |
| 2013 | 0.85 (0.64 – 1.18) | 0.24 |
| 2014 | 1.11 (0.85 – 1.45) | 0.43 |
| 2015 | 1.24 (0.96 – 1.62) | 0.10 |
| 2016 | 0.99 (0.76 – 1.29) | 0.93 |
| 2017 | 0.69 (0.52 – 0.92) | 0.01 |
| 2018 | 0.45 (0.32 – 0.63) | <0.001 |
| Referral source |  | <0.001 |
| Emergency department | 1.00 (reference) |  |
| Elective | 0.58 (0.49 – 0.69) | <0.001 |
| External hospital-referred | 0.73 (0.61 – 0.86) | <0.001 |
| Others | 0.56 (0.28 – 1.12) | 0.10 |
| Unknown | 0.73 (0.30 – 1.79) | 0.49 |
| Type of facility |  |  |
| Public | 1.00 (reference) |  |
| Private | 0.80 (0.69 – 0.92) | <0.01 |
| Complete heart block | 1.10 (0.95 – 1.28) | 0.21 |
| Sick sinus syndrome | 0.85 (0.72 – 1.002) | 0.053 |
| Acute coronary syndrome | 1.29 (0.93 – 1.80) | 0.13 |
| CABG | 1.51 (1.11 – 2.06) | <0.01 |
| All cardiac valve surgery | 3.27 (2.52 – 4.24) | <0.001 |
| TAVI | 6.20 (3.29 – 11.68) | <0.001 |
| Ischaemic heart disease | 1.19 (1.002 – 1.42) | 0.048 |
| Congestive cardiac failure | 1.34 (1.10 – 1.62) | <0.01 |
| Stroke | 1.59 (1.03 – 2.46) | 0.04 |
| Peripheral vascular disease | 1.11 (0.82 – 1.50) | 0.50 |
| Valvular heart disease | 1.49 (1.18 – 1.90) | <0.001 |
| Atrial fibrillation/flutter | 1.28 (1.11 – 1.48) | <0.001 |
| Hypertension | 1.12 (0.96 – 1.30) | 0.15 |
| Current/ex-smoker | 0.998 (0.88 – 1.13) | 0.98 |
| Malignancy | 2.99 (1.96 – 4.56) | <0.001 |
| Chronic pulmonary disease | 1.81 (1.36 – 2.41) | <0.001 |
| Chronic kidney disease | 1.27 (1.04 – 1.56) | 0.02 |
| CABG, coronary artery bypass graft; CI, confidence interval; TAVI, transcutaneous aortic valve implantation.   - Multivariable binary logistic regression method was used to identify independent predictors for all in-hospital complications; only univariables with P<0.05 were included in the multivariable analysis. | | |
