# Supplementary material for: Sex differences in rates of permanent pacemaker implantation and in-hospital complications: A statewide cohort study of over 7 million persons from 2009–2018
Source: PLoS One. 2022 Aug 10;17(8):e0272305. doi: 10.1371/journal.pone.0272305 (PMC9365143; doi:10.1371/journal.pone.0272305)
Supplement: Attachment — Submitted filename: ResponsetoReviewers.docx [file pone.0272305.s017.docx]

**PLOS ONE – Detailed Response to Editor and Reviewers’ Comments**

**Re: PONE-D-22-00216**

**Title: Sex Differences in Rates of Permanent Pacemaker Implantation and In-hospital Complications: A Statewide Cohort Study of over 7 million persons from 2009-2018**

**Journal Requirements**:

*1. When submitting your revision, we need you to address these additional requirements.*

*Please ensure that your manuscript meets PLOS ONE's style requirements, including those for file naming. The PLOS ONE style templates can be found at 
https://journals.plos.org/plosone/s/file?id=wjVg/PLOSOne_formatting_sample_main_body.pdf and* 
[*https://journals.plos.org/plosone/s/file?id=ba62/PLOSOne_formatting_sample_title_authors_affiliations.pdf*](https://journals.plos.org/plosone/s/file?id=ba62/PLOSOne_formatting_sample_title_authors_affiliations.pdf)

**Response**:	We have modified the manuscript to meet PLOS ONE’s style requirements.

2. We note that you have stated that you will provide repository information for your data at acceptance. Should your manuscript be accepted for publication, we will hold it until you provide the relevant accession numbers or DOIs necessary to access your data. If you wish to make changes to your Data Availability statement, please describe these changes in your cover letter and we will update your Data Availability statement to reflect the information you provide.

**Response**:	The New South Wales Population and Health Services Research Ethics Committee prohibits the authors from making the minimal data set publicly available. Interested researchers may contact the Ethics coordinator (ethics@cancerinstitute.org.au) to seek permission to access the data; data will then be made available upon request to all interested researchers who receive approval from the New South Wales Population and Health Services Research Ethics Committee.

We have revised the Data Availability statement in our resubmission to reflect

the above requirement to assess our study data.

**Reviewer #1**

**Major comments:**	The definition of de novo PPI should be better clarified. Is a generator replacement, if not recurrent during the index admission, included in de novo PPI? Would it be possible to differentiate new pacing system implantation from generator replacement?

I would include PPI implantation rate analysis to de novo implants only as was done for complications (simplification of the cohort and the reduction of further confounders is crucial for studies that aim to generate hypotheses).

**Response**:	Our study cohort intended to include only patients who had a new pacing system implantation and exclude patients who had recurrent procedures, including replacement of generator (which has a different ACHI procedure code to that of a new pacemaker implantation) between 1-January-2009 and 31-December-2018. We have also revised our PPI rate analysis to de novo implants as suggested.

The following changes have been made to the manuscript to clarify our definition of de novo PPI, and that the PPI rate analysis confined to de novo implants.	In the Statistical analysis section of the revised manuscript, Page 10, line 146 onwards: *“All admissions involving NSW residents between 1-January-2009 and 31-December-2018 were initially collected. The study cohort was limited to NSW residents, stratified by sex, and confined to de novo PPI to reduce confounders. For this study, de novo PPI is defined as patients who had a single PPI procedure during index admission (excluding those who had recurrent procedures or generator replacement between 1-January-2009 and 31-December-2018). This cohort was used to determine the incidence rate and temporal trend of PPI procedures.”*

In the Results section of the revised manuscript, Page 11, line 175 onwards: *“Between 1-January-2009 and 31-December-2018, there were 28,714 de novo PPI after excluding non-NSW residents (n=414) and recurrent admissions identified as admissions involving PPI (n=7,033) (S1 Fig.).”*

In the Results section under heading Incidence rate and temporal trend of PPI caseload of the revised manuscript, Page 11, line 181 onwards: *“During the 10-year study period, the mean (±SD) implantation rate was 2,871±242 cases per-annum. Though the annual volume of PPI during the study period was steady for both sexes, more men had PPI (Fig 1A). The mean total statewide population was 7,487,393±315,505 persons during the study period, with 50% women (n=3,773,756±334,912). The age-adjusted mean annual PPI was 125.5±11.6 per-100,000-men compared to 63.4±14.3 PPI per-100,000-women, P<0.01 (Fig 1B). Total volume of PPI increased exponentially beyond 40-49yo age group in both sexes (S2 Fig.). When stratified by age groups above 50yo, the rate of PPI per-100,000 in men was consistently double that of women throughout the study period, with the disparity less pronounced in age groups below 50yo (S3 and S4 Figs.). From 2009-2017 (excluding 2018 to limit ascertainment bias), the mean annual rate of increase in PPI in men was 0.9±3.3% compared to 0.4±4.4% in women (P<0.01), suggesting a widening disparity.”*.

Corresponding changes were made to the Abstract to reflect the above changes.

**Minor comments:**	In the results paragraph of the abstract, lines 37: (±standard-deviation) move after mean PPI rate lines 35

**Response**:	Change made in the revised manuscript Abstract as suggested: *“…The mean PPI rate (±standard-deviation) and median age…”.*

**Reviewer #2**

In this manuscript, the authors aim to evaluate a potential bias in PM implantations in men vs. women as well as risk of complications in men vs. women. By using an EHR database, they observed that PM are more frequently implanted in men, but that complications are more frequent in women.
Unfortunately, the main limitation of this manuscript is that only patients with a PM implantation were analyzed, instead of patients with an indication for a PM implantation (or a certain rhythm disorder). Therefore, the conclusion of the manuscript cannot be that there is or is not a bias, as the authors cannot determine if this was done in alignment with local guidelines (e.g. men having a higher risk of having rhythm disorders requiring a PM) or against local guidelines (e.g. no PM implantation in women with a rhythm disorder requiring a PM implantation). This severely limits the relevance of the findings.

**Response**:	We acknowledge that without knowing the prevalent rates of bradyarrhythmias in our statewide population limits our interpretation of whether a true gender bias in PPM implantations exist or not. As per the discussion part of the manuscript, we reviewed existing literature that had looked at the prevalence of conduction disease in the general population to see if it could partially account for the gender differences in PPM implantation rates seen in the present study. We found a lack of large population studies on the prevalence of bradyarrhythmias that require PPI. By basing our study on a population-wide unselected cohort, we hope our study finding’s nevertheless can serve as a signal that gender differences exist across all age groups, and that it can spur further investigation and etiological characterization.

We have included the following paragraph into the Limitations section on Page 23, line 398 onwards of the revised manuscript: *“The study is also limited by unknown rates of bradyarrhythmias and conduction disease in our statewide population and this limits our interpretation of whether a true gender bias in PPI exists or driven by differential rates of conduction disease between gender that requires PPI.”*

**Reviewer #3**

In the present study the authors explored the sex-related disparities in implantation of permanent pacemaker in a large Australian cohort and concluded that women are undertreated with pacing devices, this difference appears to become wider overtime. The topic related to gender and cardiac devices is attractive, the article is well written and conclusions are reasonable.

However, there are some issues that need to be addressed:

1. The large sample size allows for more extensive adjustments, in particular considering the high number of demographic and clinical variables. I would consider to assess the association between sex and PPI after correction for most of the available parameters, this might reinforce the message of lower use of PPI in women

**Response**:	We acknowledge the Reviewer comments that it would desirable to adjust for baseline demographic and clinical variables of our study cohort against that of the denominator statewide population. However, we were only able to characterize our statewide population to sex and age, and adjusted these characteristics to reflect the higher rates of PPI in men compared to women across all age groups. It would not be possible to adjust for other demographic or clinical variables of the statewide population as these parameters are not accessible to us.

However, by virtue of our large sample size, we were able to stratify our study cohort into corresponding specific age groups by decade-year of our statewide population for each calendar year of our study period, and showed that the age-adjusted PPI rates were consistently lower for women compared to men.

2. Predictors of PPI-related complications in women vs men would be of interest and might be tested in this specific sample

**Response**:	As suggested, we have now included further analysis on predictors of PPI-related complications between women and men in the revised manuscript, and the additional results are included in the revised Supplementary Material file as Tables S7-S12.

The following paragraph has been added to the revised manuscript’s result section on Page 18, line 266 onwards: *“**We further investigated the predictors of in-hospital non-fatal complications in women and separately for men (S7-12 Tables). Similar independent predictors for increased in-hospital complications risk in both men and women were younger age, cardiac valve surgery including TAVI, valvular heart disease, atrial fibrillation and malignancy, while referred as an elective procedure was associated with decreased risk of complications for both genders. Independent predictors associated with increased risk for complications in women but not in men include complete heart block presentation, history of peripheral vascular disease and hypertension. In comparison, these characteristics were associated with increased risk for complications in men but not observed in women: concomitant CABG during PPI admission, history of ischaemic heart disease, congestive cardiac failure, stroke, chronic pulmonary disease and kidney disease.”*

3. Are there available information about the socio-economic status (instruction, income, etc)

**Response**:	Unfortunately, our APDC dataset does not contain socio-economic variables such as income or education level. However, the dataset does contain marital status and country of birth, though we have not included these data variable as we did not consider these to be of clinical relevance to the implantation of pacemakers or its complications. However, should the Reviewer feel otherwise, we will be happy to include these variables into Table 1.

4. The lower age at implantation in men vs women should be further discussed, in relation with concomitant diseases and indications.

**Response**:	The following paragraph has been added into the Discussion section under Sex differences in PPI utilisation heading, Page 19, Line 299 onwards in the revised manuscript: *“**The median age of women were about 2 years older at time of PPI compared to men in this study. Sick sinus syndrome occurs primarily in older adults,[15]* *and we noted women had a higher prevalence of sick sinus syndrome as an indication for PPI, which could partially account for the observation of women receiving a pacemaker at an older age. In addition, men are known to have a lower life expectancy (5 years earlier) compared to women,[2]* *and as shown in this present study, they also have higher prevalence of ischaemic heart disease, complete heart block and other AV block and bradycardia arrhythmias. The combination of lower life expectancy and multiple cardiovascular comorbidities could account for men receiving PPI at a younger age.”*

5. I assume that ICD/CRT-P were not considered in the present study, but it should be more clearly stated.

**Response**:	Yes, ICD/CRT-P were not considered in this study.

We have added the following statement for clarity in the revised manuscript under Methods section, Study population heading, Page 4, line 82: *“Patients who had implantable cardioverter defibrillator or cardiac resynchronization therapy pacemaker were not considered for the purpose of this study.”*
