# Supplementary material for: Sex differences in rates of permanent pacemaker implantation and in-hospital complications: A statewide cohort study of over 7 million persons from 2009–2018
Source: PLoS One. 2022 Aug 10;17(8):e0272305. doi: 10.1371/journal.pone.0272305 (PMC9365143; doi:10.1371/journal.pone.0272305)
Supplement: Attachment — Submitted filename: ResponseToReviewers.docx [file pone.0272305.s018.docx]

**PLOS ONE – Detailed Response to Editor and Reviewers’ Comments**

**Re: PONE-D-22-00216R1**

**Title: Sex Differences in Rates of Permanent Pacemaker Implantation and In-hospital Complications: A Statewide Cohort Study of over 7 million persons from 2009-2018**

**Journal Requirements:**

*Please review your reference list to ensure that it is complete and correct. If you have cited papers that have been retracted, please include the rationale for doing so in the manuscript text, or remove these references and replace them with relevant current references. Any changes to the reference list should be mentioned in the rebuttal letter that accompanies your revised manuscript. If you need to cite a retracted article, indicate the article’s retracted status in the References list and also include a citation and full reference for the retraction notice.*

**Response**:	We have gone through our reference list twice and identified that for Reference 13 (“National Center for Health Statistics. Instructions for Classifying the Underlying Cause-of-Death, ICD-10. 2008: p. 1-259”), the link provided in the original manuscript was a dead link. The new link provided is confirmed to be functional and correspond to the current reference:

13. *National Center for Health Statistics. Instructions for Classifying the Underlying Cause-of-Death, ICD-10.* 2008: p. 1-259. https://www.cdc.gov/nchs/nvss/manuals/2a-sectioni-2021.htm. Accessed June 10, 2021

**Reviewer #3**

**Comment**:	The authors have to be commended for having addressed all the concerns in a fashionable and reliable way. However, the lack of information on confounders potentially affecting the relation between gender and PPI remains a major limitation. I understand that it is related to unavailable data, but it has at least to strongly stressed in the limitation section.

**Response**:	We acknowledge the Reviewer’s concern regarding potential confounders as a major limitation. For example, the lack of information on sex-based prevalence rates of bradyarrhythmia and conduction disease at a population-level meant that we cannot exclude this variable as a potential explanation for the sex differences in PPI rates observed in the present study, which we have highlighted as an important limitation of this study. Moreover, we found scarce data available in the literature reporting on the prevalence of bradyarrhythmia at a population-level, highlighting an important area for future research. We also highlighted other important confounders not available to us that may help direct future studies, including clinical data that could potentially affect outcomes of patients receiving a PPI including body mass index, prophylactic antibiotics or anticoagulation usage, and proceduralist experience.

In the Limitation section of the revised manuscript, Page 23, line 390 onwards, *“One of the major limitations of this study was unknown sex-based rates of bradyarrhythmia and conduction disease in our statewide population that required PPI, which limits our interpretation of whether a true gender bias exists or was driven by differential rates of conduction disease between gender that requires PPI.* *We found scarce data available in the literature reporting on the prevalence of bradyarrhythmia at a population-level, and we consider this to be an important area for future research.”*

In limitation section Page 23, line 398 onwards, *“We also did not have clinical data such as body mass index, proceduralist experience, whether prophylactic antibiotic was used, or the rates of prophylaxis measures against venous thromboembolism, which could all potentially act as confounders on the relationship between sex and outcomes measured in the present study.”*
